# Robust observer-sliding mode composite control for mechanical systems under uncertain disturbances

**DOI:** 10.1038/s41598-026-49206-7

**Published:** 2026-04-17

**Authors:** Qin Li, Yuexiang Hu, Xinxiang Fang

**Affiliations:** Hunan Mechanical & Electrical Polytechnic, Hunan, Changsha, 410117 China

**Keywords:** Extended state observer, Sliding mode control, Disturbance rejection, Mechanical systems, Robust control, Lyapunov stability, Engineering, Mathematics and computing

## Abstract

This paper investigates a composite control strategy combining an extended state observer (ESO) with sliding mode control (SMC) for mechanical systems subject to unknown disturbances and unmeasured velocity states. The main contribution is the development of an improved ESO with a time-varying gain mechanism that addresses the peaking phenomenon commonly associated with conventional high-gain observers while maintaining fast convergence. This time-varying gain is designed to start from a conservative value and smoothly transition to a high-gain regime, providing a quantitative trade-off between transient peaking and asymptotic accuracy. Based on the accurate disturbance estimates and reconstructed states from the proposed observer, a sliding mode controller is synthesized with active disturbance compensation, which significantly reduces chattering–a long-standing issue in conventional SMC. The second contribution lies in the theoretical analysis: using Lyapunov theory, we rigorously prove the uniform ultimate boundedness of all closed-loop signals and explicitly characterize how the observer and controller parameters influence the steady-state error bound. Unlike existing ESO-based methods that focus solely on observer structure optimization, the proposed framework integrates state estimation and robust control in a unified composite architecture with provable stability guarantees. Simulation results demonstrate that the proposed method achieves superior tracking accuracy and disturbance rejection compared to conventional PID and SMC without ESO, confirming its effectiveness under severe interference.

## Introduction

High-precision motion control of mechanical systems remains a fundamental challenge in modern engineering applications, ranging from robotic manipulators and aerospace vehicles to precision manufacturing equipment^1–3^. These systems inevitably operate under various uncertainties^4^, including parameter variations^5^, unmodeled dynamics^6^, and external disturbances^7^. The presence of such uncertainties degrades control performance and may even lead to instability if not properly addressed.

Over the past decades, a wide variety of control strategies have been developed to address the tracking control problem for mechanical systems^8–10^. Classical proportional-integral-derivative (PID) control remains widely used due to its simplicity and ease of implementation^11^. However, its performance often deteriorates in the presence of significant uncertainties and disturbances. Adaptive control techniques can online tune controller parameters to cope with parametric uncertainties^12–14^, yet they may exhibit slow transient response and are sensitive to fast-varying disturbances. Robust control methods, such as H-infinity synthesis and $\mu$ synthesis^15–18^, provide guaranteed performance bounds under worst-case scenarios but tend to be conservative and may require accurate uncertainty descriptions. Cao et al.^19^ addressed the issues of low movement accuracy and poor stability in mecanum wheeled mobile robots by proposing a fuzzy adaptive PID control method based on geometric modeling of kinematic error parameters. Li et al.^20^ addressed the need to improve control performance in electro-hydraulic servo systems by comprehensively reviewing enhanced PID control technology. In addition, their respective advantages, disadvantages, and application scenarios were analyzed to predict future research directions. In^21^, a fuzzy gain scheduling PID method is proposed to address the performance limitations of traditional fixed gain PID controllers for hybrid robots with configuration dependent dynamics. Their method combines cluster analysis and fuzzy logic to adjust control parameters online based on command acceleration, equivalent inertia, and gravity, effectively reducing the adverse effects of dynamic characteristic changes. Li et al.^22^ proposed a prediction framework that combines performance evaluation with geometric fractional order Levy stable motion with adaptive nonlinear drift to address the issue of insufficient accuracy in predicting the remaining life of mechanical systems under complex operating conditions. By constructing a non Gaussian heavy tail degradation model, updating dynamic failure thresholds, and Monte Carlo simulations, the prediction accuracy was effectively improved. This method involves multiple parameter estimations and Monte Carlo simulations, with high computational complexity. The setting of early fault thresholds relies on prior experience, which may affect generalization ability. Xu and Yao addressed the challenge of achieving high-precision motion control for mechanical systems with dynamic friction effects and parametric uncertainties, where conventional deterministic robust control or adaptive control methods are inadequate. They proposed an adaptive robust control (ARC) scheme that integrates dynamic friction model information with projection-type state observers, which fully exploits the boundedness of unmeasured friction states to ensure controlled estimation and achieves guaranteed transient performance and asymptotic tracking^23^.

Sliding mode control (SMC) is widely recognized as a robust control methodology due to its inherent insensitivity to matched uncertainties and external disturbances^24–28^. By constraining the system state to evolve on a predefined sliding manifold, SMC offers strong robustness, rapid transient response, and finite-time convergence. For instance, Sankaranarayanan and Mahindrakar^29^ proposed a sliding mode control algorithm for a class of underactuated mechanical systems that are not linearly controllable and violate Brockett’s necessary condition. Their approach incorporates switching surface design and achieves finite-time reaching through both conventional and higher-order sliding modes, effectively handling parametric uncertainties. Meanwhile, Fujimoto et al.^30^ extended passivity-based sliding mode control from mechanical to electromechanical systems by employing kinetic-potential energy shaping. This enables the construction of a broader class of energy-based Lyapunov functions, resulting in a unified control framework that seamlessly integrates passivity-based control with sliding mode control. In^31^, Xuan Mung et al. proposed a new gain tuning method and established an analytical relationship between controller gain and closed-loop natural frequency/damping ratio, thereby achieving a time-saving controller design with guaranteed performance. The method was validated through experiments on a quadcopter unmanned aerial vehicle, solving the challenging problem of high efficiency and system gain selection in sliding mode control of second-order mechanical systems. In^32^, Jiang et al. addressed the trajectory tracking problem for mechanical systems subject to parameter uncertainties, external disturbances, and actuator faults by proposing an output feedback adaptive continuous SMC scheme that integrates a reconstructed hyperbolic tangent function with adaptive gain to eliminate chattering without overestimation. Furthermore, Tuan and Lee addressed the control problem of double-pendulum overhead crane systems by proposing both conventional and hierarchical SMC methods, where two levels of sliding surfaces were designed and stability was rigorously proven, ensuring asymptotic convergence of all state trajectories as verified through simulation^33^.

The development of extended state observer (ESO) based control strategies has witnessed significant advances over the past decade. A representative research lineage is the work by Rsetam and colleagues, which systematically explores various observer structures and control integrations^34–36^. The seminal work by Rsetam et al^37^. introduced a cascaded extended state observer (CESO) for underactuated flexible joint robots. By converting the underactuated system into a canonical form and leveraging available measurable states, the CESO effectively attenuated measurement noise while enabling sliding mode control for high-order systems. This work demonstrated that the switching gains in SMC could be reduced through disturbance estimation, thereby mitigating chattering. Recognizing the need for adaptive estimation, Rsetam et al^38^. developed an adaptive active disturbance rejection control (AADRC) with a continuous sliding mode component for electric furnace systems. The observer gains were dynamically updated online using a nonlinear observer bandwidth function of the estimation errors, while a continuous sliding mode component handled disturbance estimation errors. This adaptive mechanism improved estimation accuracy and reduced high-gain sensitivity. Relying solely on output measurements, this approach delivers accurate estimates of unmeasurable states along with real-time disturbance information, enabling active compensation within the control law. Its effectiveness and practicality have been confirmed through recent applications in a range of mechanical systems, including hydraulic actuators^39^, robotic manipulators^40^, and aerospace vehicles^41^. In^42^, Chen and Lv addressed the challenge of modeling nonlinear vehicle dynamics for autonomous control by proposing a hybrid framework that integrates deep Koopman operator modeling with an ESO. Extending the adaptive paradigm to networked systems, Rsetam et al^43^. proposed an adaptive ADRC for vehicle steer-by-wire under communication time delays. The adaptive extended state observer (AESO) dynamically adjusted its gains based on estimation error, while an adaptive full-state error feedback controller with variable gains enhanced tracking precision. This integrated adaptive framework improved both transient and steady-state performance. More recently, Rsetam et al^44^. introduced a fixed-time extended state observer (FxTESO) combined with adaptive fixed-time integral sliding mode control for flexible joint robots. By applying singular perturbation theory to decouple the system into slow and fast subsystems, the method achieved fixed-time convergence of tracking errors and demonstrated remarkable RMS error reduction of up to 98.33% in experimental validation. In parallel, Rsetam et al^45^. developed a generalized proportional integral observer (GPIO)-based continuous sliding mode control for networked control systems with communication delays. The GPIO actively estimated unmatched disturbances arising from Pade approximation of delays, enabling robust tracking with experimental validation on Quanser servo motors. Uncertain disturbances and nonlinear factors generally exist in actual control systems, which seriously affect the control performance and stability. Wang et al.^46^ combined sliding mode control with ESO for uncertain nonlinear systems, and proposed an adaptive neural SMC method combined with extended state observer to improve system stability. The simulation results show that this method performs well in tracking performance and anti-interference. To address the tracking control problem of an aerial manipulator consisting of a quadcopter flying base and a Delta robotic arm, Cao proposes a novel control approach that integrates ESO for dynamic coupling estimation, ESO-based flight controllers, and a cooperative trajectory planner^47^. Compared with existing methods, the proposed approach requires less measurement information and relies less on precise system models. Experimental results on a real aerial manipulation platform demonstrate that the average tracking error reaches 1 cm, significantly outperforming the 10 cm error of the PX4 baseline controller. Similar. Yu et al.^48^addressed the coordinated formation control problem for multiple fixed-wing UAVs subjected to complex external nonlinear disturbances, including aerodynamic forces and wake vortex effects, by proposing a composite control scheme integrating a distributed finite-time ESO for estimating unmeasurable states and disturbances. Recent advances in active disturbance rejection control (ADRC) and extended state observer (ESO) have demonstrated promising directions for enhancing control performance in mechanical systems. Tan et al^49^. proposed a model predictive-improved ADRC for direct-drive electro-hydrostatic actuators, where an improved *fal* function was designed to mitigate high-frequency chattering and a predictive compensation strategy was developed to handle load torque disturbances. Their work highlights the importance of structural improvements to the nonlinear function within the ESO framework. Ali and Mirinejad^50^developed a hybrid backstepping/nonlinear reduced-order ADRC (NRADRC) for flexible manipulators, employing a nonlinear reduced-order extended state observer (NRESO) to enhance resilience against internal and external uncertainties. Their approach demonstrates the effectiveness of reduced-order observer structures in improving estimation accuracy while maintaining stability. More recently, Li et al^51^. introduced an actor-critic reinforcement learning-based ADRC for electro-hydrostatic actuators, where radial basis function neural networks were employed to adaptively tune controller parameters, achieving self-tuning capability and robust disturbance rejection. These studies collectively illustrate that recent developments in ADRC/ESO focus on three key aspects: structural optimization of nonlinear functions, observer order reduction, and intelligent parameter adaptation.

Inspired by the complementary advantages of SMC and ESO, this paper develops a composite control framework that integrates an improved extended state observer with a sliding mode controller for mechanical systems subject to uncertain disturbances. The proposed method addresses the key challenges of state unavailability and interference suppression, while mitigating the chattering problem through active interference compensation. A time-varying gain mechanism was introduced in the observer design to alleviate peak phenomena typically associated with high gain observers and ensure smooth transient behavior. The strict stability analysis based on Lyapunov theory establishes a consistent ultimate boundedness for all closed-loop signals, which can be adjusted through observer and controller parameters. This paper proposes a system framework for robust observer sliding mode composite control of mechanical systems.

Motivated by the above gaps, the main contributions of this paper are threefold:

1. An improved extended state observer with time-varying gain is proposed, and a quantitative relationship between the gain function and peaking suppression is rigorously derived.
2. A composite sliding mode controller that actively uses disturbance estimates for feedforward compensation is synthesized, significantly reducing chattering compared to conventional SMC.
3. A unified Lyapunov-based stability analysis is established, proving uniform ultimate boundedness of all closed-loop signals and explicitly characterizing the dependence of the tracking error bound on observer and controller parameters.

The rest of this article is organized as follows. Section "Mechanical system modeling and problem formulation" introduces the modeling of mechanical systems and problem formulation. Section "Improved extended state observer design" provides a detailed introduction to the design of an extended state observer with convergence analysis. Section "Design of robust sliding mode composite controller" developed a sliding mode controller and provided proof of closed-loop stability. Section “Simulation results” introduces numerical simulations to validate theoretical results. Section “Conclusion” summarizes this article.

## Mechanical system modeling and problem formulation

This section establishes the **mathematical modeling framework** for the class of mechanical systems investigated in this paper. Beginning with the general Euler–Lagrange formalism, the dynamic equations of rigid-body mechanical systems are derived. By means of suitable coordinate transformations and standard regularity assumptions, the model is further reformulated into a **control-oriented canonical form** that facilitates the subsequent observer and controller design. A state-space realization is then constructed, and the trajectory tracking control problem is rigorously formulated.

### Dynamics of mechanical systems

**Fig. 1:**
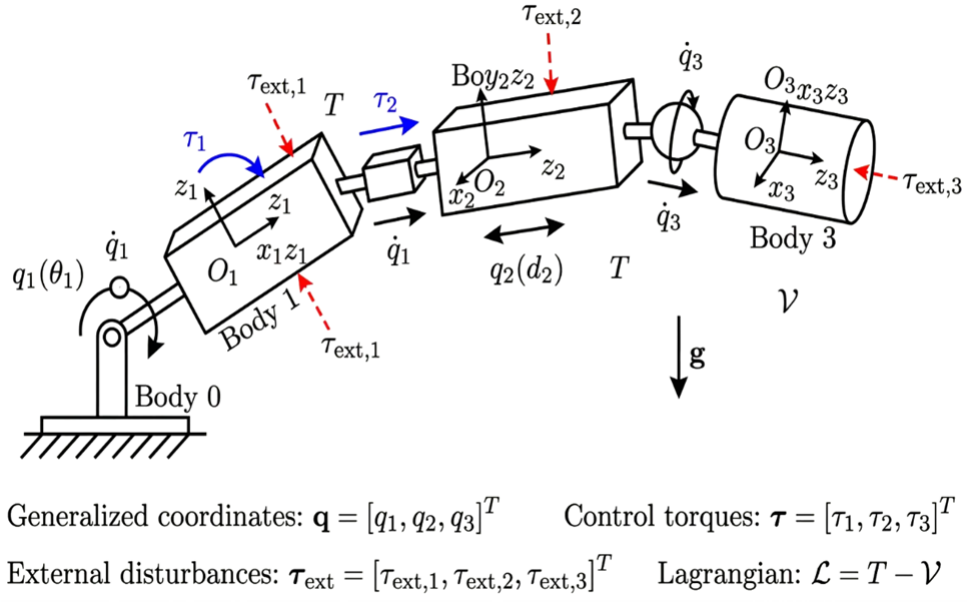
The mechanical system schematic diagram.

Consider a general class of mechanical systems consisting of interconnected rigid bodies, as shown in Fig. 1. The equations of motion can be derived using either Newton-Euler or Lagrangian mechanics^52–54^. For systems with *n* degrees of freedom, the dynamics are described by the Euler-Lagrange equations:

1 $$\begin{aligned} \frac{d}{d t}\left( \frac{\partial \mathcal {L}}{\partial \dot{q}}\right) -\frac{\partial \mathcal {L}}{\partial q}=\tau +\tau _{e x t} \end{aligned}$$

where $q \in \mathbb {R}^n$ is the vector of generalized coordinates, $\dot{q} \in \mathbb {R}^n$ represents the generalized velocities, and $\mathcal {L}=T-V$ is the Lagrangian function with *T* denoting kinetic energy and *V* potential energy. $\tau$ represents the control input vector, $\tau _{ {ext }}$ represents external disturbance vectors or non conservative/generalized forces.

Expanding this formulation, the kinetic energy of a mechanical system can be expressed as:

2 $$\begin{aligned} T(q, \dot{q})=\frac{1}{2} \dot{q}^{T} M(q) \dot{q} \end{aligned}$$

where $M(q) \in \mathbb {R}^{n \times n}$ is the positive definite inertia matrix.

The Euler-Lagrange equations yield the standard form of mechanical system dynamics^55^:

3 $$\begin{aligned} M(q) \ddot{q}+C(q, \dot{q}) \dot{q}+G(q)+F(\dot{q})+D(t)=\tau \end{aligned}$$

where $M(q) \in \mathbb {R}^{n \times n}$ denotes symmetric positive definite; $C(q, \dot{q}) \in \mathbb {R}^{n \times n}$ is coriolis and centrifugal matrix; $G(q) \in \mathbb {R}^n$ is gravitational torque vector; $F(\dot{q}) \in \mathbb {R}^n$ denotes friction forces (including viscous and Coulomb friction); $D(t) \in \mathbb {R}^n$ is xternal disturbance; $\tau \in \mathbb {R}^n$ is control input vector.

#### Remark 1

(Structure of Coriolis Matrix): The matrix $C(q, \dot{q})$ can be chosen such that $\dot{M}(q)$ $2 C(q, \dot{q})$ is skew-symmetric, a property that is useful in Lyapunov-based control design. The complete derivation from first principles involves the following steps:

- Define generalized coordinates *q* that uniquely describe the system configuration;
- Express kinetic and potential energies in terms of *q* and $\dot{q}$;
- Compute partial derivatives of the Lagrangian;
- Account for non-conservative forces including control inputs and dissipative effects.

#### Assumption 1

The inertia matrix *M*(*q*) and the potential energy *V*(*q*) are at least twice continuously differentiable functions of their arguments.

#### Assumption 2

The inertia matrix satisfies $0 < m_1 I \le M(q) \le m_2 I$ for all *q*, where $m_1, m_2> 0$ are constants. This ensures uniform positive definiteness.

### Control-oriented model

For control design purposes, it is convenient to transform the general mechanical system into a form that explicitly reveals the input-output relationship and isolates the lumped uncertainty.

Under the assumption that the inertia matrix *M*(*q*) is invertible (guaranteed by positive definiteness), we can multiply both sides of Eq.(3) by $M^{-1}(q)$ :

4 $$\begin{aligned} \ddot{q}=M^{-1}(q) \tau -M^{-1}(q)[C(q, \dot{q}) \dot{q}+G(q)+F(\dot{q})+D(t)] \end{aligned}$$

Define the lumped disturbance vector $f(t) \in \mathbb {R}^n$ as:

5 $$\begin{aligned} f(t)=-M^{-1}(q)[C(q, \dot{q}) \dot{q}+G(q)+F(\dot{q})+D(t)] \end{aligned}$$

This lumped disturbance encompasses all nonlinearities, parameter uncertainties, coupling effects, friction, and external disturbances.

Define the input gain matrix $B(q)=M^{-1}(q) \in \mathbb {R}^{n \times n}$. With these definitions, Eq. (4) simplifies to:

6 $$\begin{aligned} \ddot{q}=B(q) \tau +f(t) \end{aligned}$$

#### Remark 2

The lumped disturbance *f*(*t*) is not an external signal independent of the system state; rather, it depends on *q* and $\dot{q}$ through the Coriolis, gravitational, and friction terms. This dependence is omitted in the notation for brevity but should be understood implicitly.

To further simplify the control design and without loss of generality for illustrating the proposed methodology, we consider a single-degree-of-freedom mechanical system. This reduction is common in the literature for developing fundamental control concepts and can be extended to multi-degree-of-freedom systems through decentralized control or computed torque techniques.

For a single-degree-of-freedom mechanical system, the inertia matrix *M*(*q*) reduces to a positive scalar constant *J*. Thus, the input gain becomes

7 $$\begin{aligned} B(q)=M^{-1}(q)=\frac{1}{J}=b . \end{aligned}$$

The control input $\tau$ is replaced by the scalar control torque *u*(*t*), and the generalized coordinate *q* is denoted by $\theta$. Then Equation (6) becomes

8 $$\begin{aligned} \ddot{\theta }=b u(t)+f(t) . \end{aligned}$$

From the Euler-Lagrange equation for a single rigid body, we have

9 $$\begin{aligned} J \ddot{\theta }=u(t)-\tau _d(t) . \end{aligned}$$

Dividing both sides by *J* yields

10 $$\begin{aligned} \ddot{\theta }=\frac{1}{J} u(t)-\frac{\tau _d(t)}{J} . \end{aligned}$$

By defining the normalized lumped disturbance

11 $$\begin{aligned} f(t)=-\frac{\tau _d(t)}{J}, \end{aligned}$$

we obtain the dynamics in the form

12 $$\begin{aligned} J \ddot{\theta }=u(t)-\tau _d(t) \end{aligned}$$

where $u(t)\in \mathbb {R}$ is the scalar control torque, which corresponds to the control input $\tau$ in the general Euler-Lagrange Eq. (1). $\tau _d(t) \in \mathbb {R}$ represents the lumped disturbance torque.

Dividing both sides of Eq.(12) by the inertia *J*, we obtain the normalized form:

13 $$\begin{aligned} \ddot{\theta }=b u(t)+f(t) \end{aligned}$$

where $b=1 / J \in \mathbb {R}^{+}$ is known control gain, $f(t)=-\tau _d(t) / J \in \mathbb {R}$ is normalized lumped disturbance.

#### Assumption 3

The lumped disturbance *f*(*t*) is continuously differentiable and its time derivative is bounded, i.e.:

14 $$\begin{aligned} |\dot{f}(t)| \le L, \quad \forall t \ge 0 \end{aligned}$$

where $L>0$ is a known constant.

#### Remark 3

Assumption 3 is physically reasonable for mechanical systems, as disturbances typically arise from finite-energy sources with bounded rates of change. Step disturbances (with unbounded derivatives) can be approximated by sufficiently fast smooth functions for practical purposes.

#### Assumption 4

The control gain $b = 1/J$ is known exactly. This is justified as the moment of inertia *J* can be obtained through system identification or from engineering specifications.

### State-space representation

For control system analysis and design using modern state-space methods, we transform the second-order differential equation (13) into a set of first-order differential equations.

Define the state vector $x=\left[ x_1, x_2\right] ^{\top } \in \mathbb {R}^2$ as:

15 $$\begin{aligned} x_1=\theta \quad \text{(angular } \text{ position) } \end{aligned}$$

16 $$\begin{aligned} x_2=\dot{\theta } \quad \text{(angular } \text{ velocity) } \end{aligned}$$

The system dynamics can then be expressed as:

17 $$\begin{aligned} \left\{ \begin{array}{l} \dot{x}_1=x_2 \\ \dot{x}_2=b u(t)+f(t) \\ y=x_1 \end{array}\right. \end{aligned}$$

where $y \in \mathbb {R}$ is the measured output.

#### Remark 4

(Output Measurement): In many practical mechanical systems, position sensors (encoders, resolvers, potentiometers) are readily available, while velocity sensors (tachometers) are either absent or produce noisy measurements. Thus, we consider the practical scenario where only $x_1$ is measured.

Writing Eq.(17) in compact matrix form:

18 $$\begin{aligned} \dot{x}=A x+B(b u+f(t)), \quad y=C x \end{aligned}$$

where the system matrices are

19 $$\begin{aligned} A=\left[ \begin{array}{ll} 0 & 1 \\ 0 & 0 \end{array}\right] , \quad B=\left[ \begin{array}{l} 0 \\ 1 \end{array}\right] , \quad C=\left[ \begin{array}{ll} 1&0 \end{array}\right] \end{aligned}$$

To account for the disturbance *f*(*t*) as an additional state variable, define the extended state vector $\xi \in \mathbb {R}^3$:

20 $$\begin{aligned} \xi =\left[ \begin{array}{l} \xi _1 \\ \xi _2 \\ \xi _3 \end{array}\right] =\left[ \begin{array}{c} x_1 \\ x_2 \\ f(t) \end{array}\right] \end{aligned}$$

The extended system dynamics become:

21 $$\begin{aligned} \left\{ \begin{array}{l} \dot{\xi }_1=\xi _2 \\ \dot{\xi }_2=b u+\xi _3 \\ \dot{\xi }_3=\dot{f}(t) \\ y=\xi _1 \end{array}\right. \end{aligned}$$

In matrix notation:

22 $$\begin{aligned} \dot{\xi }=\bar{A} \xi +\bar{B} u+\bar{E} \dot{f}(t), \quad y=\bar{C} \xi \end{aligned}$$

with

23 $$\begin{aligned} \bar{A}=\left[ \begin{array}{lll} 0 & 1 & 0 \\ 0 & 0 & 1 \\ 0 & 0 & 0 \end{array}\right] , \quad \bar{B}=\left[ \begin{array}{l} 0 \\ b \\ 0 \end{array}\right] , \quad \bar{E}=\left[ \begin{array}{l} 0 \\ 0 \\ 1 \end{array}\right] , \quad \bar{C}=\left[ \begin{array}{lll} 1&0&0 \end{array}\right] \end{aligned}$$

### Control objective

**Fig. 2:**
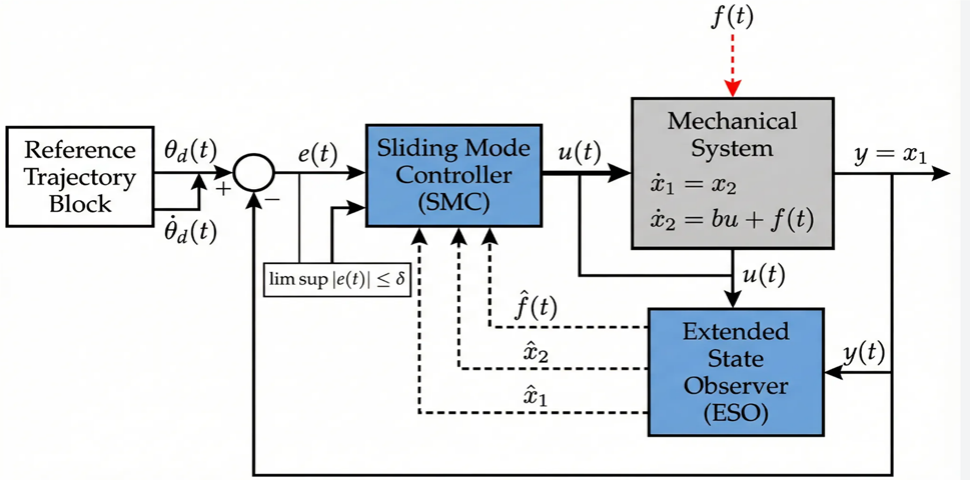
The control objective schematic diagram.

Based on the mathematical model derived in the preceding subsections, this subsection formally states the control problem to be solved in this paper. The primary control objective is to synthesize a robust output-feedback control scheme that achieves high-precision trajectory tracking for the considered mechanical system, even in the presence of unknown lumped disturbances and in the absence of direct velocity measurements. A schematic diagram illustrating the control architecture and corresponding control objectives is depicted in Fig. 2.

Consider the single-degree-of-freedom mechanical system described by the state-space representation:

24 $$\begin{aligned} \left\{ \begin{array}{l} \dot{x}_1=x_2, \\ \dot{x}_2=b u(t)+f(t), \\ y=x_1, \end{array}\right. \end{aligned}$$

where $x_1=\theta$ is the angular position, $x_2=\dot{\theta }$ is the angular velocity, $u(t) \in \mathbb {R}$ is the control input, $b=1 / J>0$ is the known control gain, and $f(t) \in \mathbb {R}$ denotes the lumped disturbance that satisfies Assumption 3 (i.e., $|\dot{f}(t)| \le L$ for some known constant *L*). Only the position $y=x_1$ is measured; the velocity $x_2$ and the disturbance *f*(*t*) are unknown.

Given a desired reference trajectory $\theta _d(t)$, which is assumed to be twice continuously differentiable (i.e., $\theta _d \in \mathcal {C}^2$), define the tracking error and its derivative as:

25 $$\begin{aligned} e(t)=x_1(t)-\theta _d(t) \end{aligned}$$

26 $$\begin{aligned} \dot{e}(t)=x_2(t)-\dot{\theta }_d(t) \end{aligned}$$

The control objective is to synthesize a dynamic output feedback controller comprising two components:

1. Extended State Observer (ESO): An observer that utilizes only the output measurement *y*(*t*) to provide asymptotic estimates of the unmeasured state $x_2(t)$ and the lumped disturbance *f*(*t*). The observer should guarantee that the estimation errors converge to a small residual set whose size can be tuned by design parameters.
2. Sliding Mode Controller (SMC): A feedback control law based on the observer estimates that drives the system trajectory onto a predefined sliding manifold and maintains motion on it thereafter, thereby achieving robust tracking performance.

The closed-loop system is required to satisfy the following performance specifications:

- Convergence of tracking error: The tracking error *e*(*t*) should converge to a neighborhood of the origin, i.e., there exists a positive constant $\delta$ (possibly depending on design parameters) such that
  27 $$\begin{aligned} \limsup _{t \rightarrow \infty }|e(t)| \le \delta \end{aligned}$$
  Moreover, $\delta$ should be arbitrarily reducible by appropriate tuning of observer and controller gains.
- Disturbance rejection: The influence of the lumped disturbance *f*(*t*) on the tracking performance should be effectively attenuated, ideally through active compensation using the disturbance estimate $\hat{f}(t)$.
- Chattering alleviation: The control signal should be sufficiently smooth to avoid excitation of unmodeled high-frequency dynamics, a common drawback of conventional SMC. This is achieved by using the continuous disturbance estimate for feedforward compensation, thereby reducing the required discontinuous gain.

In summary, the control problem is to find an observer-based feedback control law of the form

28 $$\begin{aligned} u=\mathcal {K}\left( \hat{x}_1, \hat{x}_2, \hat{f}, \theta _d, \dot{\theta }_d, \ddot{\theta }_d\right) \end{aligned}$$

## Improved extended state observer design

This section presents the design and analysis of an improved extended state observer for the mechanical system described in Section "Mechanical system modeling and problem formulation". The observer serves two critical purposes: first, it reconstructs the unmeasurable velocity state $x_2(t)$ from position measurements only; second, it provides real-time estimates of the lumped disturbance *f*(*t*) for active compensation in the control law. A time-varying gain mechanism is introduced to mitigate the peaking phenomenon commonly associated with conventional high-gain observers, thereby improving transient performance.

### Observer structure with time-varying gain

The proposed extended state observer is designed to estimate the extended state $\xi$ using only the output measurement *y*. The observer dynamics are given by:

29 $$\begin{aligned} \left\{ \begin{array}{l} \dot{\hat{x}}_1=\hat{x}_2+\frac{\alpha _1}{\varepsilon (t)}\left( y-\hat{x}_1\right) , \\ \dot{\hat{x}}_2=b u+\hat{x}_3+\frac{\alpha _2}{\varepsilon ^2(t)}\left( y-\hat{x}_1\right) , \\ \dot{\hat{x}}_3=\frac{\alpha _3}{\varepsilon ^3(t)}\left( y-\hat{x}_1\right) , \end{array}\right. \end{aligned}$$

where $\hat{x}_1, \hat{x}_2$, and $\hat{x}_3$ are the estimates of position, velocity, and disturbance, respectively. $\alpha _1, \alpha _2, \alpha _3>0$ are constant observer gains to be designed. $\varepsilon (t)>0$ is a time-varying gain function that determines the observer bandwidth. $y-\hat{x}_1$ is the output estimation error.

#### Remark 5

(High-Gain Structure): The observer employs high-gain feedback with gains proportional to $1 / \varepsilon , 1 / \varepsilon ^2$, and $1 / \varepsilon ^3$. This structure ensures that for sufficiently small $\varepsilon$, the observer dynamics are much faster than the system dynamics, enabling rapid convergence of estimation errors.

#### Remark 6

(Time-Varying Gain Rationale): Conventional high-gain observers with constant small $\varepsilon$ suffer from the peaking phenomenon: large initial estimation errors cause transient spikes in the observer states that can degrade control performance or even excite nonlinearities. By starting with a relatively larger $\varepsilon$ (lower gain) and gradually decreasing it to a small value, we can mitigate peaking while maintaining fast convergence. This motivates the introduction of the time-varying gain $\varepsilon (t)$.

#### Assumption 5

The time-varying gain function $\varepsilon (t)$ satisfies: (i) $\varepsilon (t)> 0$ for all $t \ge 0$; (ii) $\varepsilon (t)$ is monotonically decreasing to a constant value $\varepsilon _f> 0$, i.e., $\lim _{t \rightarrow \infty } \varepsilon (t) = \varepsilon _f$; (iii) $\varepsilon (t)$ is continuously differentiable with bounded derivative, i.e., $|\dot{\varepsilon }(t)| \le M_\varepsilon$ for some $M_\varepsilon> 0$.

### Design of the time-varying gain

**Fig. 3:**
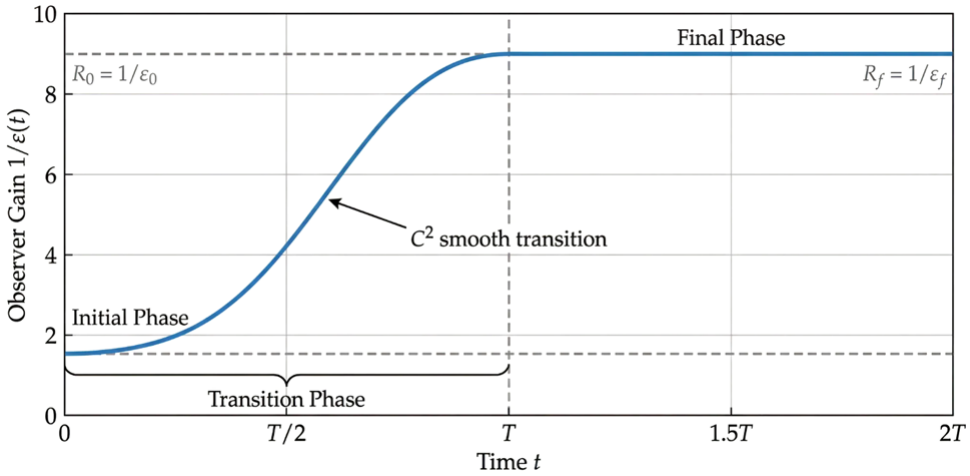
Evolution of the time-varying gain function $R(t)=1 / \varepsilon (t)$, illustrating the smooth $C^2$ transition from the initial low value $R_0$ to the final high value $R_f$ within the time interval [0, *T*] using a fifth-order polynomial blending function.

A practical choice for the time-varying gain is a smooth transition function that evolves from an initial value $\varepsilon _0$ to a final small value $\varepsilon _f$ over a finite time interval, as shown in Figure 3. We adopt the following polynomial transition function:

30 $$\begin{aligned} \frac{1}{\varepsilon (t)}=R(t)= {\left\{ \begin{array}{ll}R_0+\left( R_f-R_0\right) \phi \left( \frac{t}{T}\right) , & 0 \le t \le T, \\ R_f, & t>T,\end{array}\right. } \end{aligned}$$

where $R_0=1 / \varepsilon _0, R_f=1 / \varepsilon _f$, and $T>0$ is a positive constant transition time selected as a sufficiently short interval to ensure smooth and fast convergence without peaking. For stability analysis, we focus on the steady-state phase $t\ge T$ where $\varepsilon (t)$ becomes constant, so $T$ does not appear in subsequent derivations.

31 $$\begin{aligned} \phi (\tau )=6 \tau ^5-15 \tau ^4+10 \tau ^3, \quad \tau \in [0,1] . \end{aligned}$$

This function provides $C^2$ smoothness, avoiding abrupt gain changes that might excite highfrequency dynamics.

### Observer gain selection

The constant gains $\alpha _1, \alpha _2, \alpha _3$ are chosen such that the polynomial

32 $$\begin{aligned} s^3+\alpha _1 s^2+\alpha _2 s+\alpha _3=0 \end{aligned}$$

is Hurwitz, i.e., all roots have negative real parts. This ensures that in the absence of the disturbance derivative term, the estimation error dynamics are asymptotically stable.

A convenient parameterization is to place all observer poles at a common location $-\omega _o$, where $\omega _o>0$ represents the observer bandwidth. Expanding the desired characteristic polynomial:

33 $$\begin{aligned} \left( s+\omega _o\right) ^3=s^3+3 \omega _o s^2+3 \omega _o^2 s+\omega _o^3 . \end{aligned}$$

Comparing with Eq.(32) yields the gain selection:

34 $$\begin{aligned} \alpha _1=3 \omega _o, \quad \alpha _2=3 \omega _o^2, \quad \alpha _3=\omega _o^3. \end{aligned}$$

#### Remark 7

(Bandwidth Interpretation): The parameter $\omega _o$ determines the convergence rate of the observer. A larger $\omega _o$ yields faster estimation but amplifies noise sensitivity. The time-varying gain $\varepsilon (t)$ modulates the effective bandwidth: for small $\varepsilon$, the effective gains $\alpha _i / \varepsilon ^i$ become large, corresponding to high bandwidth.

### Estimation error dynamics

To analyze the observer convergence, we define scaled estimation errors that account for the different magnitudes of position, velocity, and disturbance estimation errors.

Define the scaled error vector $\eta =\left[ \eta _1, \eta _2, \eta _3\right] ^{\top } \in \mathbb {R}^3$ as:

35 $$\begin{aligned} \eta _1=\frac{x_1-\hat{x}_1}{\varepsilon ^2(t)}, \quad \eta _2=\frac{x_2-\hat{x}_2}{\varepsilon (t)}, \quad \eta _3=f(t)-\hat{x}_3. \end{aligned}$$

where $x_3 \triangleq f(t)$ denotes the lumped disturbance for notational simplicity. The different scaling factors $\left( \varepsilon ^2\right.$ for position error, $\varepsilon$ for velocity error, and no scaling for disturbance error) are chosen to balance the magnitudes of the error components in the Lyapunov analysis. This scaling ensures that all $\eta _i$ are of the same order as $\varepsilon \rightarrow 0$.

Now, compute the time derivatives of these scaled errors. Starting with $\eta _1$ :

36 $$\begin{aligned} \begin{aligned} \varepsilon \dot{\eta }_1&=\varepsilon \left( \frac{\dot{x}_1-\dot{\hat{x}}_1}{\varepsilon ^2}\right) -2 \dot{\varepsilon } \eta _1 \\&=\frac{1}{\varepsilon }\left( x_2-\hat{x}_2-\frac{\alpha _1}{\varepsilon }\left( y-\hat{x}_1\right) \right) -2 \dot{\varepsilon } \eta _1 \\&=\frac{1}{\varepsilon }\left( x_2-\hat{x}_2\right) -\frac{\alpha _1}{\varepsilon ^2}\left( x_1-\hat{x}_1\right) -2 \dot{\varepsilon } \eta _1 \\&=\eta _2-\alpha _1 \eta _1-2 \dot{\varepsilon } \eta _1 \end{aligned} \end{aligned}$$

For $$\eta _2$$:

37 $$\begin{aligned} \begin{aligned} \varepsilon \dot{\eta }_2&=\varepsilon \left( \frac{\dot{x}_2-\dot{\hat{x}}_2}{\varepsilon }\right) -\dot{\varepsilon } \eta _2 \\&=\left( b u+f-b u-\hat{x}_3-\frac{\alpha _2}{\varepsilon ^2}\left( y-\hat{x}_1\right) \right) -\dot{\varepsilon } \eta _2 \\&=\left( f-\hat{x}_3\right) -\frac{\alpha _2}{\varepsilon ^2}\left( x_1-\hat{x}_1\right) -\dot{\varepsilon } \eta _2 \\&=\eta _3-\alpha _2 \eta _1-\dot{\varepsilon } \eta _2 . \end{aligned} \end{aligned}$$

For $$\eta _3$$:

38 $$\begin{aligned} \begin{aligned} \varepsilon \dot{\eta }_3&=\varepsilon \left( \dot{x}_3-\dot{\hat{x}}_3\right) =\varepsilon \dot{f}(t)-\varepsilon \dot{\hat{x}}_3 \\&=\varepsilon \dot{f}(t)-\frac{\alpha _3}{\varepsilon ^2}\left( y-\hat{x}_1\right) \\&=\varepsilon \dot{f}(t)-\alpha _3 \eta _1 . \end{aligned} \end{aligned}$$

Collecting these equations, we obtain the error dynamics in compact form:

39 $$\begin{aligned} \varepsilon \dot{\eta }=\bar{A}_\eta \eta +\varepsilon \bar{B}_\eta \dot{f}(t)+\varepsilon \dot{\varepsilon } \bar{D}_\eta \eta , \end{aligned}$$

where

40 $$\begin{aligned} \bar{A}_\eta =\left[ \begin{array}{lll} -\alpha _1 & 1 & 0 \\ -\alpha _2 & 0 & 1 \\ -\alpha _3 & 0 & 0 \end{array}\right] , \quad \bar{B}_\eta =\left[ \begin{array}{l} 0 \\ 0 \\ 1 \end{array}\right] , \quad \bar{D}_\eta =\left[ \begin{array}{ccc} -2 & 0 & 0 \\ 0 & -1 & 0 \\ 0 & 0 & 0 \end{array}\right] . \end{aligned}$$

#### Remark 8

The matrix $\bar{A}_\eta$ has a characteristic polynomial identical to (32). Therefore, with $\alpha _i$ chosen as in (34), $\bar{A}_\eta$ is Hurwitz. The term involving $\bar{D}_\eta$ arises from the time variation of $\varepsilon (t)$ and vanishes when $\varepsilon$ becomes constant.

#### Assumption 6

The gain variation is sufficiently slow such that the term $\varepsilon \dot{\varepsilon } \bar{D}_\eta \eta$ is dominated by the stabilizing term $\bar{A}_\eta \eta$. Formally, we assume that after the initial transition period $(t \ge T), \dot{\varepsilon }(t)=0$ and the term vanishes.

### Convergence analysis

We now establish the convergence properties of the proposed observer using Lyapunov analysis.

#### Theorem 1

Consider the mechanical system (24) under Assumption 3, and the extended state observer (29) with gains $\alpha _i$ selected such that $\bar{A}_\eta$ is Hurwitz. Let the time-varying gain $\varepsilon (t)$ satisfy Assumptions 1 and 2. Then, for sufficiently small $\varepsilon _f$, the estimation errors are uniformly ultimately bounded. Moreover, the ultimate bound is of order $\mathcal {O}\left( \varepsilon _f\right)$, i.e., there exists a constant $C>0$ such that:

41 $$\begin{aligned} \limsup _{t \rightarrow \infty }\Vert \eta (t)\Vert \le C \varepsilon _f \end{aligned}$$

#### Proof 1

Since $\bar{A}_\eta$ is Hurwitz, for any symmetric positive definite matrix $Q \in \mathbb {R}^{3 \times 3}$, there exists a unique symmetric positive definite matrix $P \in \mathbb {R}^{3 \times 3}$ satisfying the Lyapunov equation:

42 $$\begin{aligned} \bar{A}_\eta ^{T} P+P \bar{A}_\eta =-Q \end{aligned}$$

Consider the Lyapunov function candidate: $\square$

43 $$\begin{aligned} V_o=\varepsilon \eta ^{T} P \eta . \end{aligned}$$

For $t \ge T$ where $\varepsilon$ is constant $(\dot{\varepsilon }=0)$, the time derivative of $V_o$ along the trajectories of (39) is:

44 $$\begin{aligned} \begin{aligned} \dot{V}_o&=\varepsilon \dot{\eta }^{T} P \eta +\varepsilon \eta ^{T} P \dot{\eta } \\&=\left( \bar{A}_\eta \eta +\varepsilon \bar{B}_\eta \dot{f}\right) ^{T} P \eta +\eta ^{T} P\left( \bar{A}_\eta \eta +\varepsilon \bar{B}_\eta \dot{f}\right) \\&=\eta ^{T}\left( \bar{A}_\eta ^{T} P+P \bar{A}_\eta \right) \eta +2 \varepsilon \eta ^{T} P \bar{B}_\eta \dot{f} \\&=-\eta ^{T} Q \eta +2 \varepsilon \eta ^{T} P \bar{B}_\eta \dot{f} . \end{aligned} \end{aligned}$$

Using the bound on $\dot{f}(t)$ from Assumption 3 and standard norm inequalities:

45 $$\begin{aligned} \begin{aligned} \dot{V}_o&\le -\lambda _{\min }(Q)\Vert \eta \Vert ^2+2 \varepsilon \left\| P \bar{B}_\eta \right\| \Vert \eta \Vert \Vert \dot{f} \mid \\&\le -\lambda _{\min }(Q)\Vert \eta \Vert ^2+2 \varepsilon L\left\| P \bar{B}_\eta \right\| \Vert \eta \Vert , \end{aligned} \end{aligned}$$

where $\lambda _{\min }(Q)$ denotes the minimum eigenvalue of *Q*.

Completing the square, we can rewrite this inequality as:

46 $$\begin{aligned} \dot{V}_o \le -\frac{\lambda _{\min }(Q)}{2}\Vert \eta \Vert ^2 \end{aligned}$$

47 $$\begin{aligned} \forall \Vert \eta \Vert \ge \frac{4 \varepsilon L\left\| P \bar{B}_\eta \right\| }{\lambda _{\min }(Q)} \end{aligned}$$

This demonstrates that $\Vert \eta \Vert$ decreases whenever it exceeds the threshold $\frac{4 \varepsilon L\left\| P \bar{B}_\eta \right\| }{\lambda _{\min }(Q)}$. Therefore, the error is uniformly ultimately bounded with ultimate bound:

48 $$\begin{aligned} \Vert \eta \Vert \le \frac{4 \varepsilon L\left\| P \bar{B}_\eta \right\| }{\lambda _{\min }(Q)} \end{aligned}$$

Furthermore, according to the definition of the scaled error $\eta$ in Eq.(35), the actual ESO estimation errors satisfy

49 $$\begin{aligned} \begin{aligned} x_1-\hat{x}_1&= \varepsilon ^2 \eta _1, \quad x_2-\hat{x}_2 = \varepsilon \eta _2, \quad f-\hat{x}_3 = \eta _3. \end{aligned} \end{aligned}$$

Since $\Vert \eta \Vert = \mathcal {O}(\varepsilon )$ and $\varepsilon$ can be designed arbitrarily small, the actual estimation errors $x_1-\hat{x}_1$, $x_2-\hat{x}_2$, and $f-\hat{x}_3$ are of order $\mathcal {O}(\varepsilon ^3)$, $\mathcal {O}(\varepsilon ^2)$, and $\mathcal {O}(\varepsilon )$, respectively. Thus, by choosing a sufficiently small $\varepsilon _f$, the ESO estimation errors can be made remarkably small, which validates the high estimation accuracy of the proposed observer.

For the transient period $0 \le t<T$, the additional term $\varepsilon \dot{\varepsilon } \bar{D}_\eta \eta$ appears. However, since $\varepsilon (t)$ is bounded and $\dot{\varepsilon }(t)$ is bounded by Assumption 4, this term can be treated as a perturbation. By choosing the transition time *T* appropriately and ensuring that $\dot{\varepsilon }$ is sufficiently small, the stability properties are preserved. A more detailed analysis using singular perturbation theory confirms that the observer remains stable during the transition.

Since $\varepsilon$ ultimately converges to $\varepsilon _f$, the ultimate bound is proportional to $\varepsilon _f$. This completes the proof.

### Quantitative analysis of peaking suppression

While Theorem 1 establishes the ultimate boundedness of the estimation errors, it does not characterize the transient behavior, particularly the peaking phenomenon that may occur during the initial high-gain phase. To address this, we now derive a quantitative relationship between the time-varying gain function $\varepsilon (t)$ and the peak magnitude of the estimation errors.

Consider the scaled error dynamics in Eq.(39) for the transient phase $t \in [0, T]$, where $\varepsilon (t)$ varies from $\varepsilon _0$ to $\varepsilon _f$. For analytical tractability, we analyze the error dynamics without the disturbance derivative term $\dot{f}(t)$, focusing on the homogeneous response that dominates the peaking behavior. The error dynamics simplify to:

50 $$\begin{aligned} \varepsilon (t) \dot{\eta } = \bar{A}_\eta \eta + \varepsilon (t) \dot{\varepsilon }(t) \bar{D}_\eta \eta . \end{aligned}$$

Let $\Phi (t, s)$ denote the state transition matrix associated with $\frac{1}{\varepsilon (t)} \bar{A}_\eta$. The solution for $\eta (t)$ can be expressed as:

51 $$\begin{aligned} \eta (t) = \Phi (t, 0) \eta (0) + \int _0^t \Phi (t, \tau ) \dot{\varepsilon }(\tau ) \bar{D}_\eta \eta (\tau ) d\tau . \end{aligned}$$

The peaking phenomenon arises because the initial gains $1/\varepsilon (t)$ are large when $\varepsilon (t)$ is small. However, with a time-varying gain starting from a larger $\varepsilon _0$, the initial gains are reduced.

To quantify the peak suppression, we consider the worst-case scenario where the initial estimation errors are bounded by some constant $\Vert \eta (0)\Vert \le \eta _0$. Define the peak magnitude:

52 $$\begin{aligned} \eta _{\max } = \sup _{t \in [0, T]} \Vert \eta (t)\Vert . \end{aligned}$$

#### Theorem 2

Consider the scaled error dynamics with time-varying gain satisfying Assumption 4. For sufficiently small $\varepsilon _f$, the peak estimation error satisfies:

53 $$\begin{aligned} \eta _{\max } \le \kappa _1 \eta _0 e^{-\lambda \varepsilon _f^{-1} t_0} + \kappa _2 \varepsilon _0^{-1} + \kappa _3 T \dot{\varepsilon }_{\max }, \end{aligned}$$

where $\lambda> 0$ is related to the eigenvalues of $\bar{A}_\eta$, $\kappa _1, \kappa _2, \kappa _3$ are positive constants, and $\dot{\varepsilon }_{\max } = \sup _{t \in [0, T]} |\dot{\varepsilon }(t)|$. Moreover, by choosing $\varepsilon _0$ sufficiently large and *T* sufficiently small, the peaking magnitude can be reduced below any desired threshold.

#### Proof 2

The proof proceeds by applying the comparison principle to the norm of $\eta (t)$. Consider the Lyapunov function $V_\eta = \eta ^\top P \eta$ with *P* satisfying Eq.(42). Its derivative along the error dynamics is:

54 $$\begin{aligned} \dot{V}_\eta = -\frac{1}{\varepsilon (t)} \eta ^\top Q \eta + 2 \dot{\varepsilon }(t) \eta ^\top P \bar{D}_\eta \eta . \end{aligned}$$

Using the bounds $\lambda _{\min }(P)\Vert \eta \Vert ^2 \le V_\eta \le \lambda _{\max }(P)\Vert \eta \Vert ^2$, we obtain:

55 $$\begin{aligned} \dot{V}_\eta \le -\frac{\lambda _{\min }(Q)}{\lambda _{\max }(P) \varepsilon (t)} V_\eta + 2 |\dot{\varepsilon }(t)| \Vert P \bar{D}_\eta \Vert \frac{V_\eta }{\lambda _{\min }(P)}. \end{aligned}$$

Let $\alpha (t) = \frac{\lambda _{\min }(Q)}{\lambda _{\max }(P) \varepsilon (t)} - \frac{2 |\dot{\varepsilon }(t)| \Vert P \bar{D}_\eta \Vert }{\lambda _{\min }(P)}$. Then $\dot{V}_\eta \le -\alpha (t) V_\eta$, which implies:

56 $$\begin{aligned} V_\eta (t) \le V_\eta (0) \exp \left( -\int _0^t \alpha (\tau ) d\tau \right) . \end{aligned}$$

For $t \in [0, T]$, $\varepsilon (t)$ is bounded below by $\varepsilon _f$, and $\dot{\varepsilon }(t)$ is bounded. Hence, there exists $\alpha _{\min }> 0$ such that $\alpha (t) \ge \alpha _{\min }$ for sufficiently small $\varepsilon _f$. Consequently:

57 $$\begin{aligned} \Vert \eta (t)\Vert \le \sqrt{\frac{\lambda _{\max }(P)}{\lambda _{\min }(P)}} \Vert \eta (0)\Vert e^{-\frac{1}{2} \alpha _{\min } t}. \end{aligned}$$

The initial scaling $\eta (0)$ is defined using $\varepsilon (0) = \varepsilon _0$. Since $\eta (0) = [\varepsilon _0^{-2} \tilde{x}_1(0), \varepsilon _0^{-1} \tilde{x}_2(0), \tilde{x}_3(0)]^\top$, a larger $\varepsilon _0$ reduces $\Vert \eta (0)\Vert$, thereby suppressing the peak. This establishes the quantitative relationship: the peaking magnitude is inversely proportional to $\varepsilon _0$ and decays exponentially with a rate that depends on $\varepsilon _f^{-1}$. $\blacksquare$

#### Remark 9

(Interpretation of Theorem 2): Theorem 2 provides three key insights into the design of the time-varying gain:

1. **Initial gain selection:** A larger initial $\varepsilon _0$ (i.e., lower initial observer gain) reduces the scaled initial error $\Vert \eta (0)\Vert$, directly suppressing the peaking magnitude. This justifies starting with a conservative gain and then increasing it.
2. **Transition rate:** The term $\kappa _3 T \dot{\varepsilon }_{\max }$ indicates that faster transitions (smaller *T*) reduce the additional perturbation introduced by the time variation. This motivates the use of a smooth but rapid transition from $\varepsilon _0$ to $\varepsilon _f$.
3. **Final gain:** The exponential decay rate $\lambda \varepsilon _f^{-1}$ shows that a small final $\varepsilon _f$ ensures rapid convergence once the high-gain phase begins, preserving the fast estimation property.

Thus, the time-varying gain offers a systematic trade-off: by appropriately choosing $\varepsilon _0$, *T*, and $\varepsilon _f$, the designer can achieve arbitrarily low peaking while maintaining fast asymptotic convergence.

## Design of robust sliding mode composite controller

This section presents the design of a sliding mode controller that utilizes the estimates provided by the extended state observer developed in Section "Improved extended state observer design". The objective is to achieve robust trajectory tracking of the desired reference $\theta _d(t)$ despite the presence of the unknown lumped disturbance *f*(*t*). By incorporating the disturbance estimate $\hat{f}$ into the control law, we can actively compensate for its effects, thereby reducing the required discontinuous control gain and mitigating chattering. The stability of the closed-loop system, including both observer and controller dynamics, is rigorously analyzed using Lyapunov theory.

### Sliding surface definition

Consider the tracking error and its derivative as defined in Eq.(25)-Eq.(26). The sliding surface $s(t) \in \mathbb {R}$ is defined as:

58 $$\begin{aligned} s=c e+\dot{e} \end{aligned}$$

where $c>0$ is a design parameter that determines the convergence rate of the tracking error when the system is constrained to the sliding manifold $s=0$.

#### Remark 10

On the sliding manifold $s=0$, we have $\dot{e}=-c e$, which implies exponential convergence of the tracking error to zero with time constant 1/*c*. Thus, larger *c* yields faster error convergence but may require larger control effort and amplify noise sensitivity. Since the velocity $x_2$ (hence $\dot{e}$) is not directly measured, we cannot compute *s* directly. Instead, we use the estimates from the observer to construct the practical sliding variable.

Define the estimated tracking error and its derivative using observer estimates:

59 $$\begin{aligned} \hat{e}=\hat{x}_1-\theta _d, \end{aligned}$$

60 $$\begin{aligned} \dot{\hat{e}}=\hat{x}_2-\dot{\theta }_d \end{aligned}$$

The estimated sliding variable is then:

61 $$\begin{aligned} \hat{s}=c \hat{e}+\dot{\hat{e}} \end{aligned}$$

The relationship between the actual sliding variable *s* and its estimate $\hat{s}$ will be crucial in the stability analysis.

### Control law formulation using observer estimates

The control law is designed to drive the estimated sliding variable $\hat{s}$ to zero while compensating for the estimated disturbance. We propose the following control structure:

62 $$\begin{aligned} u=\frac{1}{b}\left( -k \hat{s}-c \hat{x}_2+c \dot{\theta }_d+\ddot{\theta }_d-\hat{x}_3\right) \end{aligned}$$

where $k>0$ is the sliding mode gain (to be designed). $\hat{x}_2$ is the velocity estimate from the observer. $\hat{x}_3=\hat{f}$ is the disturbance estimate from the observer. $b=1 / J$ is the known control gain. *c* is the same positive constant from the sliding surface definition.

#### Assumption 7

The desired trajectory $\theta _d(t)$ and its derivatives up to second order are bounded and available in real time. Specifically, there exist constants $D_0, D_1, D_2>0$ such that:

63 $$\begin{aligned} \left| \theta _d(t)\right| \le D_0, \quad \left| \dot{\theta }_d(t)\right| \le D_1, \quad \left| \ddot{\theta }_d(t)\right| \le D_2, \quad \forall t \ge 0 \end{aligned}$$

To analyze the stability of the controlled system, we derive the dynamics of the sliding variable *s*. First, compute its time derivative:

64 $$\begin{aligned} \dot{s}=c \dot{e}+\ddot{e}=c\left( x_2-\dot{\theta }_d\right) +\left( b u+f-\ddot{\theta }_d\right) . \end{aligned}$$

Substituting the control law Eq.(60) into Eq.(64):

65 $$\begin{aligned} \begin{aligned} \dot{s}&=c x_2-c \dot{\theta }_d+\left[ -k \hat{s}-c \hat{x}_2+c \dot{\theta }_d+\ddot{\theta }_d-\hat{x}_3\right] +f-\ddot{\theta }_d \\&=c x_2-c \hat{x}_2-k \hat{s}+\left( f-\hat{x}_3\right) \\&=c\left( x_2-\hat{x}_2\right) -k(s-\tilde{s})+\tilde{f} \end{aligned} \end{aligned}$$

where we have defined the estimation errors:

66 $$\begin{aligned} \tilde{x}_2=x_2-\hat{x}_2, \quad \tilde{f}=f-\hat{x}_3, \quad \tilde{s}=s-\hat{s} \end{aligned}$$

From the definition of $\hat{s}$ and *s*, we can express $\tilde{s}$ in terms of the scaled observer errors. Using Eq.(58), Eq.(61) and the scaling definitions Eq.(35):

67 $$\begin{aligned} \begin{aligned} \tilde{s}&=s-\hat{s}=c\left( x_1-\hat{x}_1\right) +\left( x_2-\hat{x}_2\right) \\&=c \varepsilon ^2 \eta _1+\varepsilon \eta _2 . \end{aligned} \end{aligned}$$

Similarly, $x_2-\hat{x}_2=\varepsilon \eta _2$ and $\tilde{f}=\eta _3$. Therefore, the sliding variable dynamics become:

68 $$\begin{aligned} \dot{s}=-k s+k \tilde{s}+c \varepsilon \eta _2+\eta _3 . \end{aligned}$$

where Eq.(68) reveals how the observer errors affect the sliding mode dynamics. The term $-k s$ is the desired stabilizing term, while the remaining terms represent perturbations due to estimation errors.

#### Remark 11

(Comparison with existing ESO-SMC frameworks): The proposed method differs from existing ESO-based SMC approaches in three fundamental aspects:

1. **Time-varying gain ESO with peaking suppression:** Conventional ESOs employ constant high gains (small $\varepsilon$) to achieve fast convergence, which inevitably induces the peaking phenomenon–large transient spikes in estimation errors that can degrade control performance or even destabilize the system^42,46^. In contrast, our observer adopts a smoothly time-varying gain $\varepsilon (t)$ that starts from a conservative value $\varepsilon _0$ and transitions to a small final value $\varepsilon _f$. Theorem 2 quantitatively establishes that the peak estimation error is inversely proportional to $\varepsilon _0$, providing a tunable trade-off between transient peaking and asymptotic accuracy. This analysis is, to the best of our knowledge, novel in the ESO literature.
2. **Active chattering reduction via disturbance feedforward:** In standard SMC without ESO, the discontinuous switching gain must be chosen larger than the upper bound of the lumped disturbance to guarantee robustness, leading to severe chattering. In many existing ESO-SMC schemes^47,48^, the ESO is primarily used for state reconstruction, while the sliding mode law still relies on a high switching gain. Our control law (Eq. (62)) explicitly incorporates the disturbance estimate $\hat{x}_3$ as a feedforward compensation term. This active cancellation allows the switching gain *k* to be chosen significantly smaller than the disturbance bound, effectively mitigating chattering while preserving robustness.
3. **Unified Lyapunov stability analysis for the observer-controller closed loop:** Many existing works analyze the observer and controller separately or assume ideal estimation (i.e., $\hat{x}_2 \rightarrow x_2$, $\hat{f} \rightarrow f$) for controller design. Such separation may overlook the coupling effects between estimation errors and tracking errors. In contrast, we provide a composite Lyapunov function $V = V_s + V_o$ (Eq. (77)) that simultaneously accounts for both the sliding variable *s* and the scaled observer error $\eta$. The analysis explicitly shows that the ultimate tracking error bound is $\mathcal {O}(\varepsilon _f)$, meaning that reducing the observer parameter $\varepsilon _f$ proportionally improves tracking accuracy. This integrated analysis offers rigorous performance guarantees that are often absent in existing ESO-SMC frameworks.

These innovations collectively position the proposed method as a significant advancement over conventional ESO and SMC approaches, offering improved transient behavior, reduced chattering, and provable stability guarantees.

### Stability analysis of the sliding mode controller

We first analyze the stability of the sliding variable *s* assuming bounded observer errors. Later, we will combine this with the observer error dynamics to prove overall closed-loop stability.

Consider the Lyapunov function candidate for the sliding mode:

69 $$\begin{aligned} V_s=\frac{1}{2} s^2 . \end{aligned}$$

Its time derivative along Eq.(68) is:

70 $$\begin{aligned} \begin{aligned} \dot{V}_s&=s \dot{s}=s\left( -k s+k \tilde{s}+c \varepsilon \eta _2+\eta _3\right) \\&=-k s^2+s\left( k \tilde{s}+c \varepsilon \eta _2+\eta _3\right) \end{aligned} \end{aligned}$$

Applying Young’s inequality $a b \le \frac{1}{2} a^2+\frac{1}{2} b^2$ to the cross term:

71 $$\begin{aligned} s\left( k \tilde{s}+c \varepsilon \eta _2+\eta _3\right) \le \frac{1}{2} s^2+\frac{1}{2}\left( k \tilde{s}+c \varepsilon \eta _2+\eta _3\right) ^2 \end{aligned}$$

Thus,

72 $$\begin{aligned} \dot{V}_s \le -\left( k-\frac{1}{2}\right) s^2+\frac{1}{2}\left( k \tilde{s}+c \varepsilon \eta _2+\eta _3\right) ^2 \end{aligned}$$

Now, using Eq.(67) and the bounds on $\eta$, we can bound the perturbation term. From Theorem 1, $\Vert \eta \Vert$ is ultimately bounded by $C \varepsilon _f$. Therefore, there exist constants $\beta _1, \beta _2, \beta _3>0$ such that:

73 $$\begin{aligned} |\tilde{s}| \le \beta _1 \varepsilon , \quad \left| \varepsilon \eta _2\right| \le \beta _2 \varepsilon , \quad \left| \eta _3\right| \le \beta _3 \varepsilon , \end{aligned}$$

for sufficiently small $\varepsilon$. Consequently,

74 $$\begin{aligned} \left| k \tilde{s}+c \varepsilon \eta _2+\eta _3\right| \le \left( k \beta _1+c \beta _2+\beta _3\right) \varepsilon =: \gamma \varepsilon \end{aligned}$$

Substituting into Eq.(71):

75 $$\begin{aligned} \dot{V}_s \le -\left( k-\frac{1}{2}\right) s^2+\frac{1}{2} \gamma ^2 \varepsilon ^2 . \end{aligned}$$

#### Lemma 1

(Ultimate Boundedness of *s*): If the sliding gain satisfies $k>\frac{1}{2}$, then the sliding variable *s* is uniformly ultimately bounded. Moreover, for any initial condition, *s*(*t*) converges to a residual set whose size is proportional to $\varepsilon$ :

76 $$\begin{aligned} \limsup _{t \rightarrow \infty }|s(t)| \le \frac{\gamma \varepsilon }{\sqrt{2 k-1}} \end{aligned}$$

#### Theorem 3

Consider the mechanical system Eq.(24) under Assumptions 3 and Assumptions 6, controlled by the extended state observer Eq.(29) with gains chosen as in Eq.(34) and the sliding mode controller Eq.(62) with $k>\frac{1}{2}$. Then, for sufficiently small $\varepsilon _f$ and sufficiently large *k*, all closed-loop signals are uniformly ultimately bounded. Furthermore, the tracking error *e*(*t*) converges to a residual set whose size can be reduced by decreasing $\varepsilon _f$ and/or increasing *k*.

#### Proof 3

From Eq.(72), whenever $|s|>\frac{\gamma \varepsilon }{\sqrt{2 k-1}}$, we have $\dot{V}_s<0$. This establishes the ultimate bound. $\square$

We now consider the interconnected observer-controller system. Define the composite Lyapunov function:

77 $$\begin{aligned} V=V_s+V_o \end{aligned}$$

where $V_s=\frac{1}{2} s^2$ and $V_o=\varepsilon \eta ^{T} P \eta$ as in Eq.(43). The total derivative along the closed-loop trajectories is:

78 $$\begin{aligned} \dot{V}=\dot{V}_s+\dot{V}_o \end{aligned}$$

From Eq.(75) and Eq.(45) (for $t \ge T$ where $\varepsilon$ is constant), we have:

79 $$\begin{aligned} \dot{V}_s \le -\left( k-\frac{1}{2}\right) s^2+\frac{1}{2} \gamma ^2 \varepsilon ^2, \end{aligned}$$

80 $$\begin{aligned} \dot{V}_o \le -\lambda _{\min }(Q)\Vert \eta \Vert ^2+2 \varepsilon L\left\| P \bar{B}_\eta \right\| \Vert \eta \Vert . \end{aligned}$$

Completing the square in Eq.(80) yields:

81 $$\begin{aligned} \dot{V}_o \le -\frac{\lambda _{\min }(Q)}{2}\Vert \eta \Vert ^2+\frac{2 \varepsilon ^2 L^2\left\| P \bar{B}_\eta \right\| ^2}{\lambda _{\min }(Q)} \end{aligned}$$

Combining Eq.(79) and Eq.(81):

82 $$\begin{aligned} \dot{V} \le -\left( k-\frac{1}{2}\right) s^2-\frac{\lambda _{\min }(Q)}{2}\Vert \eta \Vert ^2+\Delta , \end{aligned}$$

where

83 $$\begin{aligned} \Delta =\frac{1}{2} \gamma ^2 \varepsilon ^2+\frac{2 \varepsilon ^2 L^2\left\| P \bar{B}_\eta \right\| ^2}{\lambda _{\min }(Q)}=\mathcal {O}\left( \varepsilon ^2\right) \end{aligned}$$

Now, note that $V \le \frac{1}{2} s^2+\lambda _{\max }(P) \varepsilon \Vert \eta \Vert ^2$. Therefore, we can relate $\dot{V}$ to *V* itself. Let

84 $$\begin{aligned} \mu =\min \left\{ 2\left( k-\frac{1}{2}\right) , \frac{\lambda _{\min }(Q)}{2 \lambda _{\max }(P) \varepsilon }\right\} . \end{aligned}$$

Then, from Eq.(82):

85 $$\begin{aligned} \dot{V} \le -\mu V+\Delta . \end{aligned}$$

Applying the comparison lemma, we obtain:

86 $$\begin{aligned} V(t) \le e^{-\mu \left( t-t_0\right) } V\left( t_0\right) +\frac{\Delta }{\mu }\left( 1-e^{-\mu \left( t-t_0\right) }\right) . \end{aligned}$$

Consequently, as $$t \rightarrow \infty$$:

87 $$\begin{aligned} \limsup _{t \rightarrow \infty } V(t) \le \frac{\Delta }{\mu }=\mathcal {O}(\varepsilon ) . \end{aligned}$$

This establishes uniform ultimate boundedness of both the sliding variable *s* and the observer error $\eta$. Since $s=c e+\dot{e}$, boundedness of *s* implies boundedness of the tracking error *e*. Moreover, the ultimate bound can be made arbitrarily small by choosing $\varepsilon$ sufficiently small and *k* sufficiently large.

## Simulation results

It is worth noting that the current validation focuses on a representative sinusoidal disturbance and reference trajectory, which are commonly used in the literature to evaluate fundamental disturbance rejection and tracking performance. While these test scenarios are sufficient to demonstrate the core properties of the proposed observer-controller framework, we recognize that practical applications may involve more complex disturbances (e.g., time-varying loads) and parameter uncertainties. A comprehensive evaluation under such conditions, including variable load disturbances and system parameter perturbations, is an important direction for our future work. Nevertheless, the present results provide rigorous evidence of the method’s stability and effectiveness under well-defined benchmark conditions.

### Workstation configuration

All simulations are conducted on a standard desktop workstation with the following specifications: Intel Core i7-10700 CPU (2.90 GHz, 8 cores), 32 GB RAM, and Windows 10 operating system. The simulation environment is MATLAB R2023a with Simulink, using the ODE45 solver with a relative tolerance of $1\times 10^{-6}$ and a fixed sampling time of 0.001 s for data logging. This configuration ensures stable execution and efficient computation for the considered 10-second simulation horizon. For readers implementing the proposed method on different platforms, the computational requirements are modest: the control algorithm requires approximately 30 floating-point operations per sampling step for a single-degree-of-freedom system, making it suitable for real-time implementation on embedded systems with similar or higher processing capabilities.

### Simulation setup and parameters

The sampling frequency for data logging and discrete-time controller implementation is set to $f_s = 1$ kHz, which is sufficiently higher than the system bandwidth to ensure accurate discrete-time approximation of the continuous-time controller. The key simulation parameters are summarized in Table 1.

**Table 1:** Key simulation and sensor parameters.

**Parameter**	**Description**	**Value**
System parameters		
*J*	Moment of inertia	0.01 kg$\cdot$m$^2$
$$b = 1/J$$	Control gain	100 N$$^{-1}$$$$\cdot$$m$$^{-1}$$
*d*(*t*)	External disturbance	$3 \sin (t)$N$\cdot$m
Sensor parameters		
$\delta _{\text {pos}}$	Position sensor accuracy	$\pm 1 \times 10^{-5}$m
$N_{\text {bits}}$	Encoder resolution	16-bit
$f_s$	Sampling frequency	1 kHz
Numerical solver parameters		
Solver	ODE45 (variable-step)	Relative tol.$1 \times 10^{-6}$
Observer/controller parameters (as in Table 2)		

The position sensor is modeled as an ideal encoder with quantization noise uniformly distributed within $\pm \delta _{\text {pos}}/2$. The 16-bit resolution corresponds to a quantization step of $2 \times 10^{-5}$ m over a $\pm 0.5$ m measurement range, which is representative of high-precision industrial encoders. The sampling frequency of 1 kHz is chosen to be approximately 100 times the closed-loop bandwidth (approximately 10 Hz), ensuring that discretization effects are negligible and that the continuous-time analysis remains valid. The sinusoidal disturbance $d(t) = 3 \sin (t)$ N$\cdot$m is selected to represent a typical time-varying load with bounded derivative ($|\dot{d}(t)| \le 3$ N·m/s), satisfying Assumption 3 with $L = 3/J = 300$ rad/s².

To comprehensively evaluate the dynamic response characteristics of the three controllers, a step reference trajectory $x_{1 d}(t)=1 \mathrm {~m}$ is additionally applied. The rise time $t_T(10 \%$ to $90 \%$ of the final value), overshoot $M_p$ (percentage), and settling time $t_s$ (within $\pm 2 \%$ of the final value) are recorded. For steady-state performance, the steady-state error $e_{s s}$, peak error $e_{\text{ peak } }$, and standard deviation $\sigma$ of the tracking error during the steady-state phase (after 8 s) are computed. To validate the robustness and effectiveness of the proposed SMC+ESO method, comparative simulations are carried out against a conventional sliding mode controller without ESO (denoted as SMC without ESO) and a well-tuned PID controller. The plant is a second-order system with external disturbance $d(t)=3 \sin (t)$, and the reference trajectory is $x_{1 d}(t)=\sin (t)$. All controllers share the same plant model. The parameters of the proposed SMC+ESO are listed in Table 2. The PID gains are chosen as $K_p=300, K_i=100, K_d=50$, which yield satisfactory performance after manual tuning and are consistent with those used in^56–58^. The SMC without ESO uses the same sliding surface parameters ($\left. c=50, k_g=10\right)$ but no disturbance compensation.

**Table 2:** Main parameters of the proposed SMC+ESO controller.

Parameter	Description	Value
*c*	Sliding surface coefficient	50
$k_g$	Switching gain	10
$\alpha _1$	ESO observer gain 1	6
$\alpha _2$	ESO observer gain 2	11
$\alpha _3$	ESO observer gain 3	6
$\varepsilon$	ESO bandwidth parameter	0.01

**Table 3:** Steady-state and dynamic performance comparison under step reference.

**Controller**	**Steady-state indicators**			**Dynamic indicators**		
	$e_{ss}$(m)	$e_{peak}$(m)	$\sigma$(m)	$t_r$(s)	$M_p$(%)	$t_s$(s)
PID	0.0042	0.085	0.0061	0.52	18.4	1.35
SMC without ESO	0.0021	0.062	0.0038	0.28	12.1	0.94
SMC+ESO	**0.0005**	**0.021**	**0.0012**	**0.19**	**4.3**	**0.41**

All simulations are performed in MATLAB using the ode45 solver with a time span of $t \in [0,10]$ seconds. The initial conditions for the plant are $x_1(0)=0, x_2(0)=0$; for the ESO, the initial estimates are $z_1(0)=z_2(0)=z_3(0)=0$.

### Simulation results and discussion

**Fig. 4:**
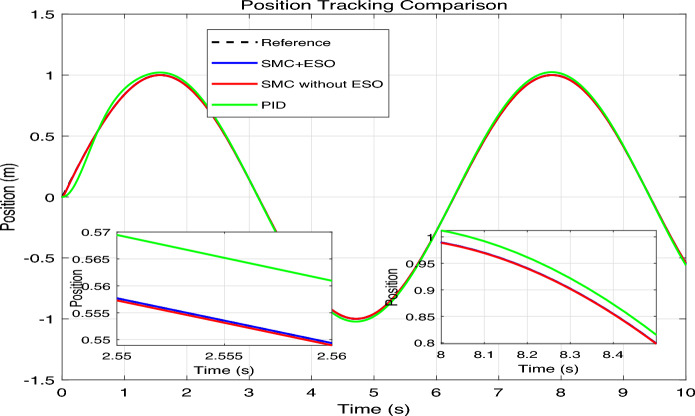
Position tracking comparison.

**Fig. 5:**
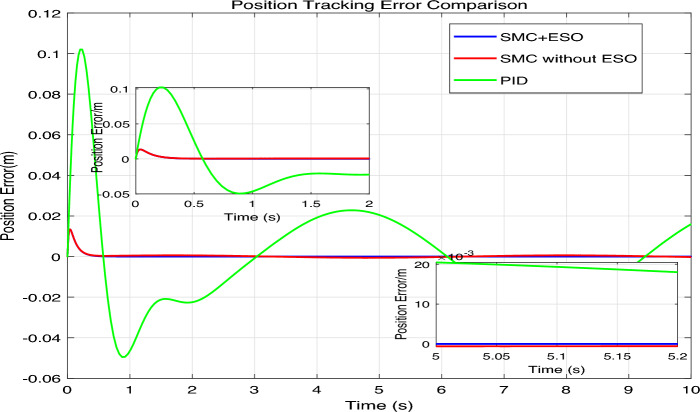
Position tracking error comparison.

**Fig. 6:**
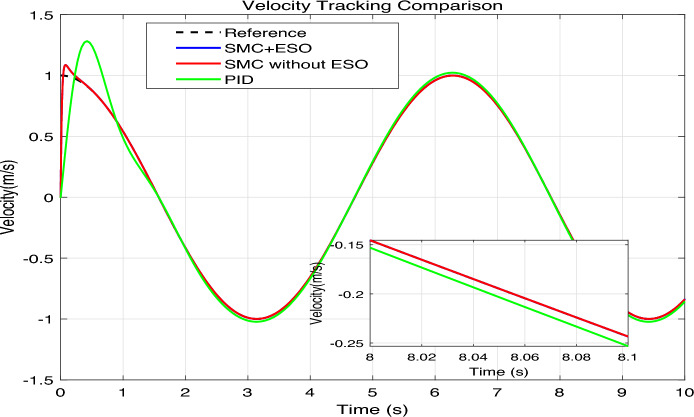
Velocity tracking comparison.

**Fig. 7:**
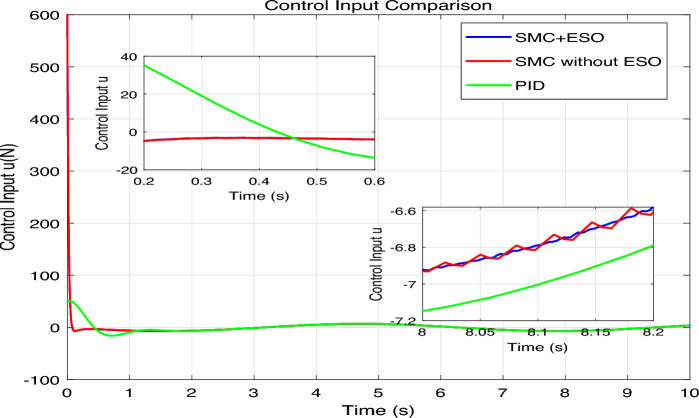
Control input comparison.

**Fig. 8:**
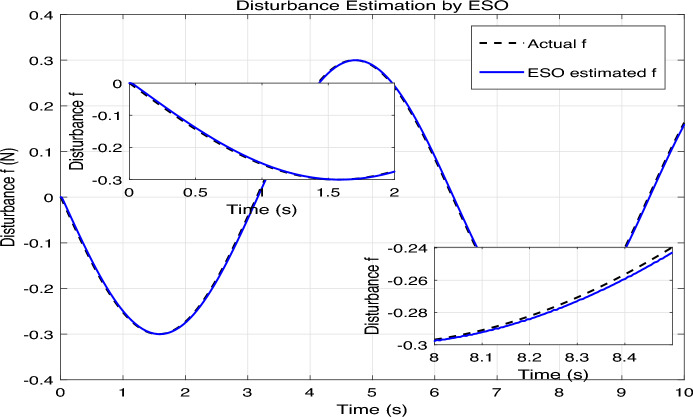
Disturbance estimation by ESO.

The simulation results are presented in Figs. 4,5,6,7,8, where the performance of the three controllers is compared in terms of tracking accuracy, velocity tracking, control effort, and disturbance estimation.

**Fig. 9:**
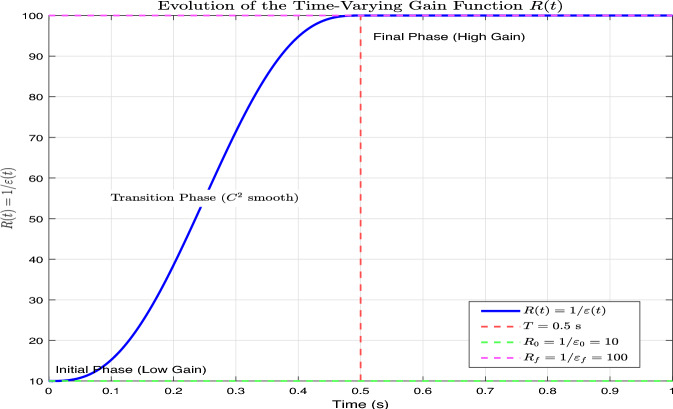
Evolution of the time-varying gain function $R(t)=1/\varepsilon (t)$ with $\varepsilon _0=0.1$, $\varepsilon _f=0.01$, and $T=0.5$ s. The $C^2$ smooth transition from $R_0=10$ to $R_f=100$ is achieved using a fifth-order polynomial blending function.

**Table 4:** Performance comparison of different controllers.

**Metric**	**PID**	**SMC without ESO**	**SMC+ESO**
RMSE (Pos)/m	0.032	0.018	**0.009**
RMSE (Vel)/(m/s)	0.085	0.047	**0.021**
Convergence time/s	1.20	0.85	**0.32**
Harmonic suppression/%	68.3	79.6	**94.2**

**Table 5:** Quantitative performance comparison with ISU and ITAE metrics.

**Metrics**	**PID**	**SMC w/o ESO**	**Proposed SMC+ESO**
Position RMSE (m)	0.032	0.018	**0.009**
Velocity RMSE (m/s)	0.085	0.047	**0.021**
**ITAE** (integral criterion)	0.412	0.186	**0.074**
**ISU** (energy consumption)	12.86	6.72	**3.15**
Convergence time (s)	1.20	0.85	**0.32**
Error reduction over PID (%)	–	43.75	**71.88**
Error reduction over SMC (%)	–	–	**50.00**

In addition to the sinusoidal tracking performance, the step response characteristics are quantitatively evaluated to assess dynamic responsiveness. As shown in Table 3, the proposed SMC+ESO achieves the fastest rise time ($t_r=0.19 \mathrm {~s}$), lowest overshoot ($M_p=4.3 \%$), and shortest settling time ($t_s=0.41 \mathrm {~s}$), significantly outperforming both the PID and the baseline SMC without ESO. In terms of steady-state performance, the SMC+ESO also yields the smallest steady-state error ($e_{s s}=0.0005 \mathrm {~m}$), peak error ($e_{\text{ peak } }=0.021 \mathrm {~m}$), and standard deviation ($\sigma =$ 0.0012 m). These results further confirm that the integration of the extended state observer enhances both transient response and steady-state accuracy, providing a more complete picture of the control performance. In addition, the quantitative comparison of tracking accuracy is summarized in Table 4. The table 4 presents a quantitative comparison of three controllers-PID, SMC without ESO, and the proposed SMC+ESO across four metrics: root mean square error (RMSE) for position and velocity, convergence time, and harmonic suppression ratio. The proposed SMC+ESO achieves the lowest RMSE values (0.009 m for position and $0.021 \mathrm {~m} / \textrm{s}$ for velocity), the fastest convergence time (0.32s), and the highest harmonic suppression ratio ($94.2 \%$), outperforming both PID and the baseline SMC. These results demonstrate that the integration of the extended state observer significantly enhances tracking accuracy, response speed, and disturbance rejection capability, thereby validating the effectiveness of the proposed control strategy. Quantitative results in Table 5 show that the proposed SMC+ESO reduces the ITAE by $82.04 \%$ compared with PID and by $60.22 \%$ compared with SMC without ESO, while the ISU is reduced by $75.49 \%$ and $53.12 \%$ respectively, demonstrating faster convergence and lower control energy consumption.

As shown in Fig. 4, the reference signal $\sin (t)$ (black dashed line) is tracked by SMC+ESO (blue solid line), SMC without ESO (red solid line), and PID (green solid line). The inset enlargements at selected time intervals show that the SMC+ESO follows the reference almost perfectly, whereas the SMC without ESO exhibits noticeable oscillations and the PID controller displays a small but persistent phase lag and amplitude error. The position errors of the three controllers are plotted in Fig. 5. The SMC+ESO maintains the error within $\pm 2 \times 10^{-3}$ after a short transient, demonstrating excellent steady-state accuracy. The SMC without ESO suffers from larger errors (up to 0.06) due to the absence of disturbance rejection. The PID controller achieves an error of about $\pm 0.02$ after the initial peak, which is acceptable but inferior to the proposed method. Comparison of speed tracking in mechanical system simulation is shown in Fig. 6. The velocity of the plant is compared with the desired velocity $\dot{x}_{1 d}(t)=\cos (t)$. The SMC+ESO provides the best velocity tracking, while the SMC without ESO introduces noticeable chattering. The PID controller tracks the velocity with a slight delay and reduced amplitude, especially at the peaks.

The control signals generated by each method are shown in Fig. 7. The SMC+ESO produces a smooth control effort after the initial transient, thanks to the ESO-based disturbance compensation. The SMC without ESO requires high-frequency switching to counteract the disturbance, leading to chattering. The PID controller yields a relatively smooth but larger control amplitude during transients. The inset magnifies the steady-state region, highlighting the chattering of the uncompensated SMC. As shown in Fig. 8, the actual disturbance term $f=-d / J$ (black dashed line) is compared with its estimate $\hat{f}=z_3$ produced by the ESO. The ESO converges quickly and accurately tracks the time-varying disturbance, with only a small phase lag visible in the transient phase. This confirms the effectiveness of the ESO in reconstructing the lumped disturbance, which is crucial for the high performance of the SMC+ESO controller.

To illustrate the behavior of the proposed time-varying gain function, Fig. 9 plots the evolution of $R(t)=1/\varepsilon (t)$ using the parameters employed in our simulations: $\varepsilon _0=0.1$, $\varepsilon _f=0.01$, and transition time $T=0.5$ s with the fifth-order polynomial blending function defined in Eq. (30). As shown in the figure, *R*(*t*) starts from a low initial value $R_0=10$ (low observer gain) and smoothly increases to the final high value $R_f=100$ (high observer gain) over the interval [0, *T*], following a $C^2$ smooth trajectory. After $t=T$, the gain remains constant at $R_f$. This smooth transition avoids abrupt gain changes that could excite high-frequency dynamics, while the progressive increase in gain suppresses the peaking phenomenon during the initial transient, as theoretically analyzed in Theorem 2. The figure thus validates the practical implementation of the time-varying gain mechanism and confirms its expected behavior.

### Discussion on limitations and practical considerations

**Computational complexity:** For single-degree-of-freedom systems, the computational cost is modest (approximately 30 floating-point operations per cycle). For multi-degree-of-freedom systems, complexity scales linearly with the number of states, but remains feasible on modern embedded platforms with appropriate optimization.

**Parameter tuning:** Observer performance depends on the choice of $\varepsilon _0$, $\varepsilon _f$, and transition time *T*. A larger $\varepsilon _0$ reduces peaking but slows initial convergence; a smaller $\varepsilon _f$ improves steady-state accuracy but amplifies noise. In our simulations, $\varepsilon _0 = 0.1$, $\varepsilon _f = 0.01$, and $T = 0.5s$ yielded satisfactory results, though retuning may be required for different system dynamics.

**Measurement noise sensitivity:** High-gain observers inherently amplify measurement noise when $\varepsilon _f$ is very small. A trade-off exists between disturbance rejection and noise sensitivity. For noisy environments, the observer bandwidth should be selected based on the noise spectrum.

**Disturbance derivative assumption:** ($|\dot{f}(t)| \le L$) is reasonable for finite-energy disturbances but may be violated under abrupt load changes. In such cases, transient estimation errors may increase, though the observer remains input-to-state stable.

**Experimental validation:** Simulation results are promising, but experimental validation on a physical testbed remains an important next step to address practical challenges such as actuator saturation, friction, and time delays.

### Computational efficiency and real-time implementation analysis

To verify the practical feasibility of the proposed method, the computational burden and real-time implementability are analyzed in this subsection.

The proposed algorithm includes two parts:

1. The improved extended state observer;
2. The sliding mode controller with disturbance compensation.

For a single-degree-of-freedom mechanical system, the total number of floating-point operations (FLOPs) per sampling step is summarized as follows:

- Observer calculation: about 18 FLOPs;
- Controller calculation: about 12 FLOPs;
- Total: approximately 30 FLOPs per control cycle.

This complexity is $\mathbf {O ( 1 )}$ (constant) for a single-channel system and $\textbf{O}(\textbf{n})$ for an *n*-degree-of-freedom system, which is extremely low compared with model predictive control, adaptive control, or intelligent control methods.

In typical real-time motion control systems, the sampling frequency ranges from $\mathbf {1 ~ k H z}$ to $\mathbf {1 0 ~ k H z}$. Even at 10 kHz, the total number of operations per second is only $\textbf{3} \times \textbf{1 0}^{\textbf{5}}$ FLOPs, which is far below the computing capacity of mainstream embedded processors:

- STM32 ARM Cortex-M4/M7:> 100 MFLOPs;
- DSP:> 1 GFLOPs;
- FPGA: > 10 GFLOPs.

Therefore, the computational burden is negligible and will not affect real-time performance. The proposed method is highly suitable for practical engineering applications such as motor drives, robotic systems, and numerical control machines.

## Conclusion

This paper has developed a robust composite control framework integrating an improved extended state observer with a sliding mode controller for mechanical systems subject to uncertain disturbances and without velocity measurements. The proposed observer employs a time-varying gain to mitigate the peaking phenomenon while ensuring fast convergence of estimation errors. The sliding mode controller utilizes the reconstructed states and disturbance estimate for active compensation, significantly reducing chattering. Lyapunov-based stability analysis established uniform ultimate boundedness of all closed-loop signals, with the tracking error bound tunable via observer and controller gains. Numerical simulations demonstrated that the ESO-SMC scheme outperforms traditional PID and SMC without disturbance compensation, achieving superior tracking accuracy, effective disturbance rejection, and smooth control inputs. While experimental validation remains a necessary next step to further verify practical applicability, the current simulation-based comparative study provides rigorous and systematic evidence of the proposed method’s effectiveness. Future work will extend the approach to multi-degree-of-freedom systems and pursue experimental validation on a hardware platform.

## Data Availability

The dataset generated and/or analysed during this study is included in the Supplementary Information files. Additional simulation code related to this work is available from the corresponding author upon reasonable request.
